# Acetylcholinesterase Inhibition Reverses Age-Related Pulmonary Decline and Increases Bronchus-Associated Lymphoid Tissue Formation in Aged Mice

**DOI:** 10.3390/biology15030270

**Published:** 2026-02-03

**Authors:** Kyle Kenny, Ingrid R. Niesman, Kee S. Moon, Mark Sussman, Morgan K. Wright, Dylan Dawood, Joy A. Phillips

**Affiliations:** 1 Department of Biology, San Diego State University, San Diego, CA 92182, USA; kdkenny19@gmail.com (K.K.); msussman@sdsu.edu (M.S.);; 2 Department of Mechanical Engineering, San Diego State University, San Diego, CA 92182, USA; kmoon@sdsu.edu; 3 SDSU Integrated Regenerative Research Institute, San Diego, CA 92182, USA; 4 Department of Chemistry and Biochemistry, San Diego State University, San Diego, CA 92182, USA; ddawood6541@sdsu.edu

**Keywords:** inflammaging, cholinergic tone, acetylcholine, donepezil, iBALT, lung function, aging, tissue-resident memory, collagen, elastin

## Abstract

Age-related chronic, low-grade inflammation, known as inflammaging, contributes to tissue damage and disease. In the lungs, inflammaging leads to abnormal tissue remodeling, reduced function, and decreased immunity. A key factor in inflammaging is declining acetylcholine signaling, which normally suppresses inflammation and promotes tissue repair. We tested whether increasing acetylcholine responsiveness could reverse age-related lung damage. Aged mice were treated with donepezil to increase acetylcholine availability. After six months, blood oxygen saturation and voluntary activity were significantly improved. Histologically, treated mice showed a reversal of alveolar enlargement (a hallmark of emphysema) and complete restoration of elastic fibers. Donepezil treatment also dramatically increased bronchus-associated lymphoid tissue (iBALT) formation. iBALT is the repository of tissue-resident memory lymphocytes, including memory cholinergic lymphocytes that produce acetylcholine to suppress inflammation during secondary infections. The age-related loss of iBALT contributes to the increased risks associated with respiratory infection in the elderly. This indicates that age-related lung function and respiratory immune deficits can be modulated by improving acetylcholine signaling. Repurposing an approved medication provides a direct pathway to clinical application for improving respiratory health and infection resistance during aging.

## Simple Summary

As people age, their lungs and immune system do not work as well as they used to. This makes older adults much more vulnerable to pneumonia, flu, and other breathing illnesses. Scientists know that levels of a natural chemical called acetylcholine, which helps control inflammation, drop with age, and that raising acetylcholine in older adults is linked to a lower risk of death. This study tested whether donepezil (an Alzheimer’s drug that increases acetylcholine) could improve lung and immune health in aged mice. Older mice that received donepezil were more active and had better blood oxygen levels, more elastic lungs, and healthier immune tissue in their lungs than untreated aged mice. These results suggest that a drug already approved for older patients might one day help protect lung function and immune defenses in people with age-related or chronic breathing problems.

## 1. Introduction

The lung maintains optimal gas exchange conditions through its extensive alveolar surface area [1,2]. Structural integrity of alveolar units depends on the pulmonary extracellular matrix (ECM), composed of elastic fibers and collagen, providing the tissue with rigidity and elasticity essential for efficient respiration [3]. Elastic recoil of alveolar septa is a fundamental property for normal lung function, maintained through precise cell signaling and elastase expression [4,5]. Alveolar fibrin deposition is another indicator of respiratory distress or possible acute lung injury [6]. The lung is also a major site of pathogen exposure; thus, the mucosal immune system must mediate protective inflammatory responses while limiting damage to the delicate alveolar architecture.

The lungs undergo significant structural and functional deterioration with advancing age in response to the chronic, low-grade inflammatory state termed “inflammaging” [1,2,7,8,9]. Inflammaging leads to pathologic remodeling of the ECM, resulting in reduced elasticity and decreased lung function [6,10,11,12,13]. Key alterations include increased airspace volume, enhanced proteolysis, reduced alveolar branching complexity, and decreased capillary density [14,15,16,17]. This pathological airspace enlargement, termed “senile pulmonary emphysema,” is associated with measurable declines in pulmonary function, resulting in decreased activity tolerance and reduced quality of life [18,19,20,21,22]. Inflammaging also negatively impacts the mucosal immune response [18,23,24]. As a result, respiratory infections disproportionately increase morbidity and mortality in elderly populations [25,26,27].

The key feature of inflammaging is chronic inflammatory cytokine production. Cytokine-mediated inflammation is regulated by acetylcholine (ACh) in a brain-immune circuit termed the cholinergic anti-inflammatory pathway (CAP) [28,29,30]. ACh and non-neuronal cholinergic signals are both hotly studied topics at the leading edge of biology. Recent research has shown that ACh has extremely diverse functionality outside of the nervous system [31,32,33]. From the regulation of pulmonary epithelial cell behavior to the immune cell activation of resident tissue macrophages and lymphocytes, the cholinergic system is much broader in scope than is widely recognized [34,35,36]. Many immune cells in the body possess the correct receptors, enzymes, and other components required for production and responsiveness to ACh, indicating that immune system function may be intimately tied to the overall cholinergic tone of the organism [37,38,39]. Cholinergic tone, or production of and responsiveness to ACh, decreases with aging, directly corresponding to the increased inflammatory cytokine production found in inflammaging [40,41,42]. Increasing cholinergic tone in the elderly results in decreased systemic inflammation, improved clinical outcomes following respiratory viral infection, and reduced all-cause mortality [43,44,45,46]. This suggests the effects of inflammaging could be mitigated by increased production of and/or response to ACh [43,47,48,49,50,51,52,53,54,55,56].

These observations led us to hypothesize that enhancing cholinergic tone might reverse the deleterious effects of aging/inflammaging on lung structure and function. To test this hypothesis, aged mice were treated with the acetylcholinesterase (AChE) antagonist donepezil to increase ACh bioavailability. Treated mice displayed higher voluntary running, increased blood oxygen saturation, reduced alveolar size, decreased overt inflammation, and increased elastic fiber content relative to age-matched controls. Treated mice also had larger quantities of induced bronchus-associated lymphoid tissue (iBALT). These findings are completely novel and indicate that enhancing cholinergic tone may present a promising therapeutic avenue to improve lung structure, function, and immune responsiveness during aging.

## 2. Materials and Methods

Animal Studies: Male and female C57BL/6 mice were bred in-house and used in all studies. Animal experimental protocols were approved by the Institutional Animal Care and Use Committee (IACUC) at San Diego State University before initiation of experiments. Animals were given free access to food and water and were cared for according to guidelines set by the American Association for Laboratory Animal Care. Aged C57BL/6 male and female mice (18–24 mo.) were administered 2.5 mg/kg/day in the drinking water (based on intake volume of 5 mL/mouse/day) for 6 months. Additional lungs from young male and female C57BL/6 mice (4 mo.) were obtained from the NIA repository.

Voluntary Wheel Running (VWR): Mice were individually housed in a cage containing a disk-style running wheel (Innovive Products, San Diego, CA, USA) with a magnet attached to the back of the disk, detectable via a magnetic inductive proximity switch counter. Mice had normal access to food and water during the trial, with each trial lasting 18 h. Animals rested overnight between trials. Each animal had at least four separate running trials prior to donepezil treatment to determine baseline activity. Post-treatment VWR was compared to pre-treatment VWR for each animal.

Specific Blood Oxygen Measurement: A novel prototype pulse oximeter was used to measure oxygen saturation (SpO_2_). The wired reflectance pulse oximeter was fitted inside a half-inch “Mini-Hair Claw Clip” (Hotop via Amazon.com, Seattle, WA, USA), which was placed around the rib cage. Mice were acclimatized to the device by wearing the clip several times prior to analysis. The day before observation, animals had their chest, stomach, and abdomen shaved to ensure proper contact between the sensor and the skin. For measurement, each animal was lightly sedated with isoflurane to allow optimal sensor placement for pulse oximetry measurement. Testing started when animals had regained consciousness and were ambulatory. Each animal was tested twice; each test ran for 100 s. Data were analyzed in MATLAB version R2020b. SpO_2_ values lower than 70 were considered erroneous and removed.

Histology: Lungs were inflated with 10% buffered formalin using the fixed-volume method, then fixed in 10% buffered formalin for 24 h, and finally dehydrated in 100% ethanol [57]. The samples were embedded in paraffin according to standard protocols for microtomy, and lungs were serially sectioned at 5 µm [58]. Sections were processed for visualization and quality control via Hematoxylin and Eosin stain [59]. A Modified Russell Movat Pentachrome stain was used to quantify elastic fibers (black) and fibrin (scarlet) [60]. Collagen and iBALT were quantified via differential staining with Sirius Red and Fast Green, as described [61]. Slides were imaged using a BZ-X810 Fluorescence Microscope (Keyence Corp., Osaka, Japan). Images were captured with the 10× lens (100× total power), and exposure at 1/800. Image tiles were then combined into a single large file of the whole tissue section for analysis.

**Figure A1:**
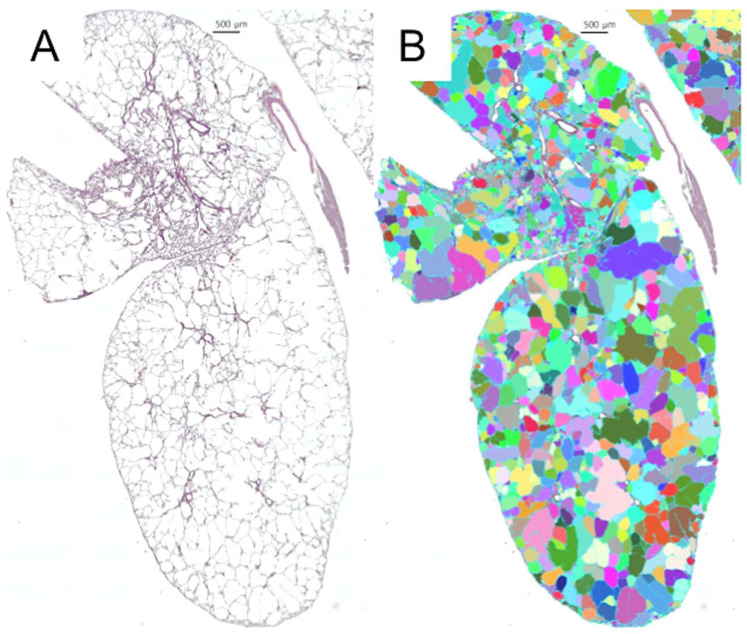
Representative lung demonstrating Mean Alveolar Area analysis. (**A**) Aged murine lung stained with Hematoxylin and Eosin. (**B**) Falsely colored representation of the alveolar area produced by the cell counter. Slides were imaged using the Keyence BZ-X810: composite scan, magnification 100×, and scale 500 µm.

Image Analysis: After capture, the images were given a 500-micron scale bar for comparison. Mean alveolar area (MAA) was quantified using the Keyence Hybrid Cell Count software program (version BZ-H4C). See Appendix A for a more detailed methodology. MAA was derived from section-based morphometry after fixed-volume inflation; therefore, values may be influenced by shrinkage and orientation (Figure A1). Each lung sample was analyzed for the percentage of tissue area (%) that stained positive for elastic fibers, fibrin, collagen, or iBALT. Thresholds for analysis were applied uniformly across cohorts; the extracted pixel area is reported as the percentage of tissue area (%). Section-level sampling was balanced across cohorts, and section analysis was averaged per individual animal. Identification of iBALT was based on morphology and location; immunophenotypic confirmation was not performed.

Statistical Analysis: All quantitative values are expressed as the mean ± standard error or mean ± standard deviation, as stated. Standard error was utilized for comparisons between the groups to assess the precision of our mean estimates. Standard deviation was utilized to determine the biologic variability within the samples. Cohorts were compared via one-way ANOVA and Tukey’s post hoc HSD or via one-tailed *t*-test. Treatment groups were all assessed for sex-specific differences compared to the mean. Since none were identified, the male and female populations were pooled for analysis. All one-way ANOVAs, Tukey’s HSD tests, one-tailed *t*-tests, and additional descriptive statistics were performed with GraphPad Prism version 9.0 for Windows (GraphPad Software, Boston, MA, USA). Further, *p*-values ≤ 0.05 were considered significant.

## 3. Results

### 3.1. Donepezil Treatment Preserves Physical Activity and Oxygenation in Aged Mice

**Figure 1:**
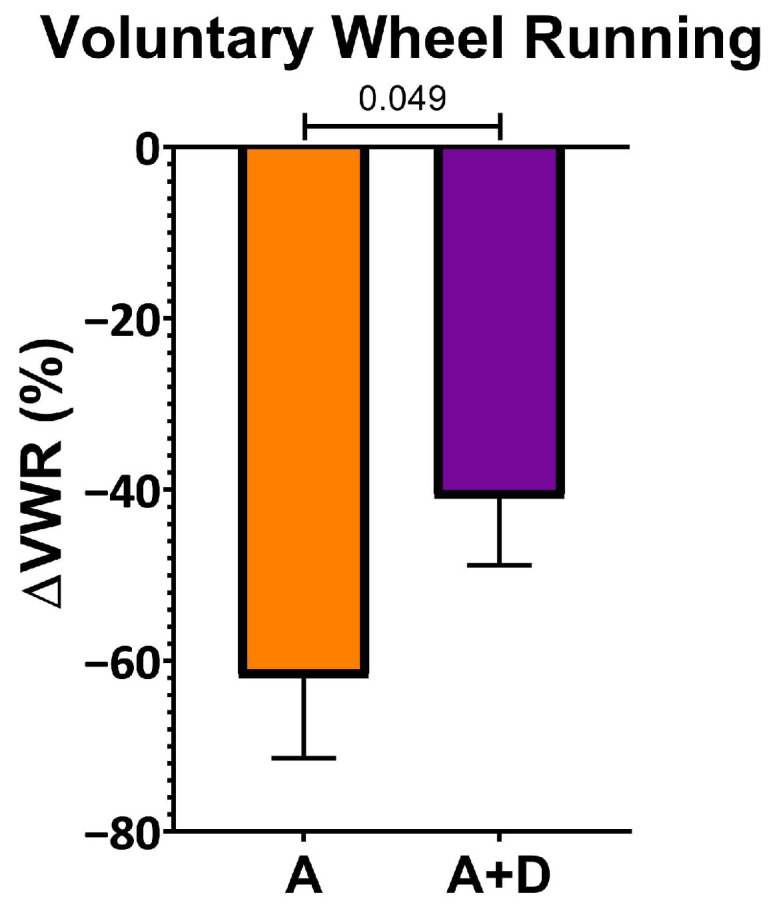
*Donepezil treatment preserves voluntary activity*. Percentage change in voluntary wheel running between age 12 months (baseline) and 24 months (post-treatment). Aged untreated mice (A) showed greater activity decline than aged mice treated with donepezil for 6 months (A + D). Mouse n = 6–8 per group. Bars represent the mean ± SEM; *p* values shown in the figure were calculated using one-tailed *t*-test.

To assess the functional impact of donepezil treatment on healthspan, we first measured spontaneous activity using a VWR assay, a sensitive indicator of overall health in mice. Baseline activity was established at 12 months of age, after which donepezil treatment began at 18 months and continued for 6 months, with a final VWR assessment at 24 months. As expected, both aged groups showed substantial declines in voluntary activity by 24 months of age (Figure 1). However, the decline was significantly attenuated in donepezil-treated mice (−41 ± 22%) compared to untreated aged controls (−62 ± 23%, *p* < 0.05). This preservation of approximately one-third of the age-related activity decline suggests that enhanced cholinergic signaling maintains physical capacity during aging.

### 3.2. Donepezil Treatment Improves Blood Oxygen Saturation

**Figure 2:**
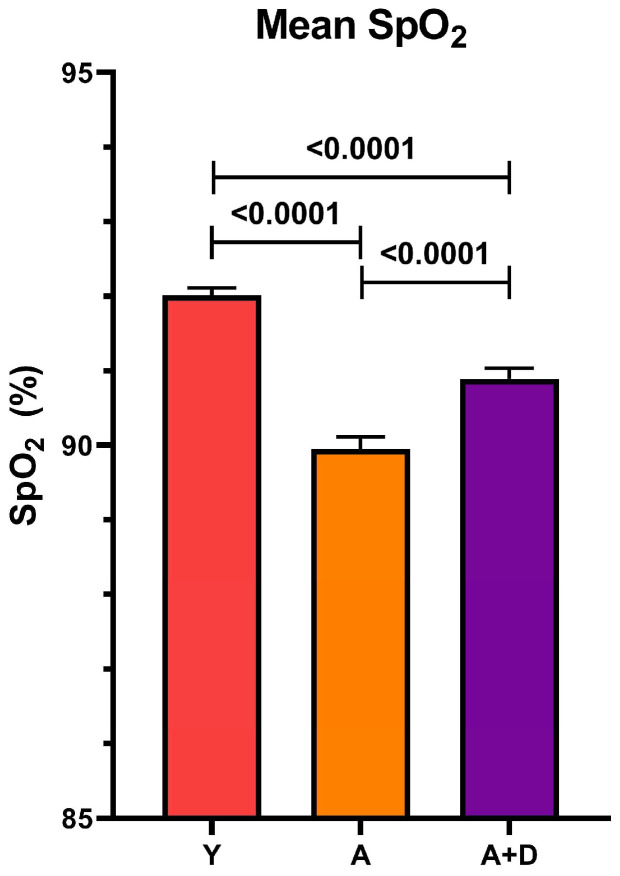
*Donepezil treatment improves blood oxygenation in aged mice.* SpO_2_ in young (Y), aged untreated (A), and aged donepezil-treated (A + D) mice. Mouse n = 3–4 per group. Bars represent mean ± SEM; *p* values shown in the figure were calculated using one-way ANOVA with Tukey’s post hoc HSD.

We next measured peripheral capillary oxygen saturation (SpO_2_) at 24 months using reflectance pulse oximetry, a non-invasive assessment of pulmonary gas exchange efficiency and cardiovascular function. One-way ANOVA revealed significant differences between groups (F_2,1851_ = 53.59, *p* < 0.0001; Figure 2). Young controls exhibited the highest SpO_2_ (92 ± 2%), with all pairwise comparisons reaching statistical significance (*p* < 0.05). Critically, donepezil-treated aged mice (91 ± 4%) maintained oxygenation levels intermediate between young controls and aged untreated animals (90 ± 4%). While the absolute difference of 1% may appear modest, even small improvements in SpO_2_ can reflect meaningful enhancement of pulmonary function and tissue oxygenation, particularly in the context of age-related physiological decline. Together with the preserved activity levels, these findings suggest that donepezil treatment maintains integrated cardiopulmonary function during aging.

### 3.3. Histological Assessment of Age-Related Pulmonary Changes

**Figure 3:**
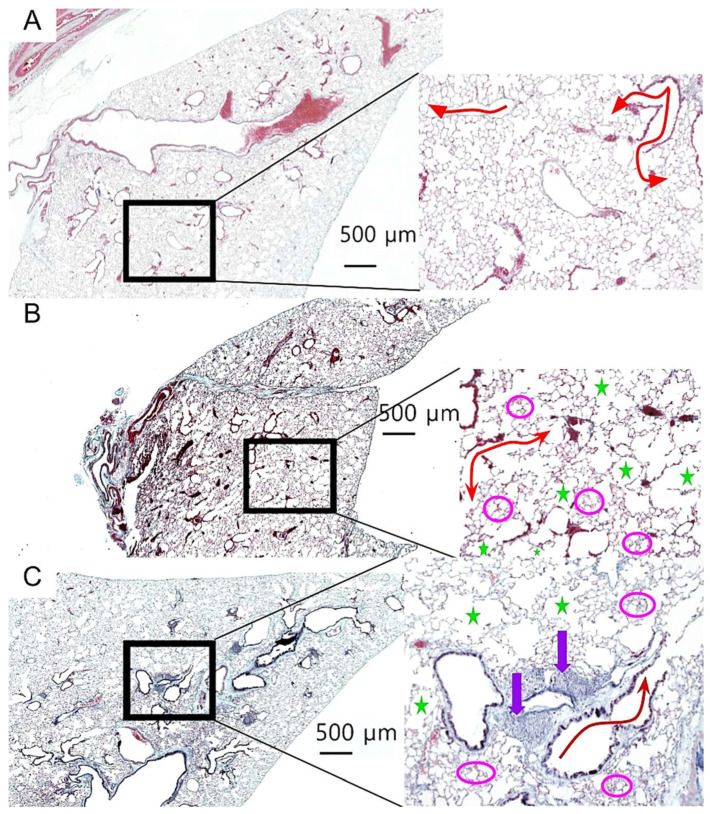
*Age-related changes to lung architecture.* Lung sections stained with Modified Russell Movat Pentachrome: Representative photomicrographs of (**A**) young lung (age 4 months; n = 6). (**B**) Aged control (24 months; n = 28). (**C**) Aged donepezil-treated lungs (24 months; n = 20). Magnified images at right show evidence of age-related pulmonary pathology. Red arrow = alveolar tubule; green star = emphysematic alveoli; fuchsia circles = fibrin; purple arrow = iBALT. Imaged using a Keyence BZ-X810. Scale bar 500 µm, magnification 100×.

To assess the effects of aging and donepezil treatment on lung architecture, we performed a comprehensive histological analysis of lung tissue sections. Representative images from each cohort are shown in Figure 3, with the magnified insets highlighting key architectural features. Young control lungs displayed normal alveolar architecture with uniform airspace size and intact extracellular matrix, with no histological evidence of age-related pathology (Figure 3A). In contrast, both aged cohorts exhibited classical features of pulmonary aging, including alveolar enlargement, focal emphysematous changes, and fibrin deposition (Figure 3B,C). Notably, donepezil-treated aged mice displayed prominent iBALT follicles, characterized by dense lymphocytic aggregates along the bronchi (Figure 3C).

To quantify these observed changes, we measured the mean alveolar area to assess airspace enlargement, fibrin deposition as a marker of chronic tissue injury, elastic fiber content to evaluate extracellular matrix integrity, and collagen distribution to distinguish pathological fibrosis from iBALT-associated stromal changes.

#### 3.3.1. Donepezil Treatment Partially Preserves Alveolar Architecture in Aged Lungs

**Figure 4:**
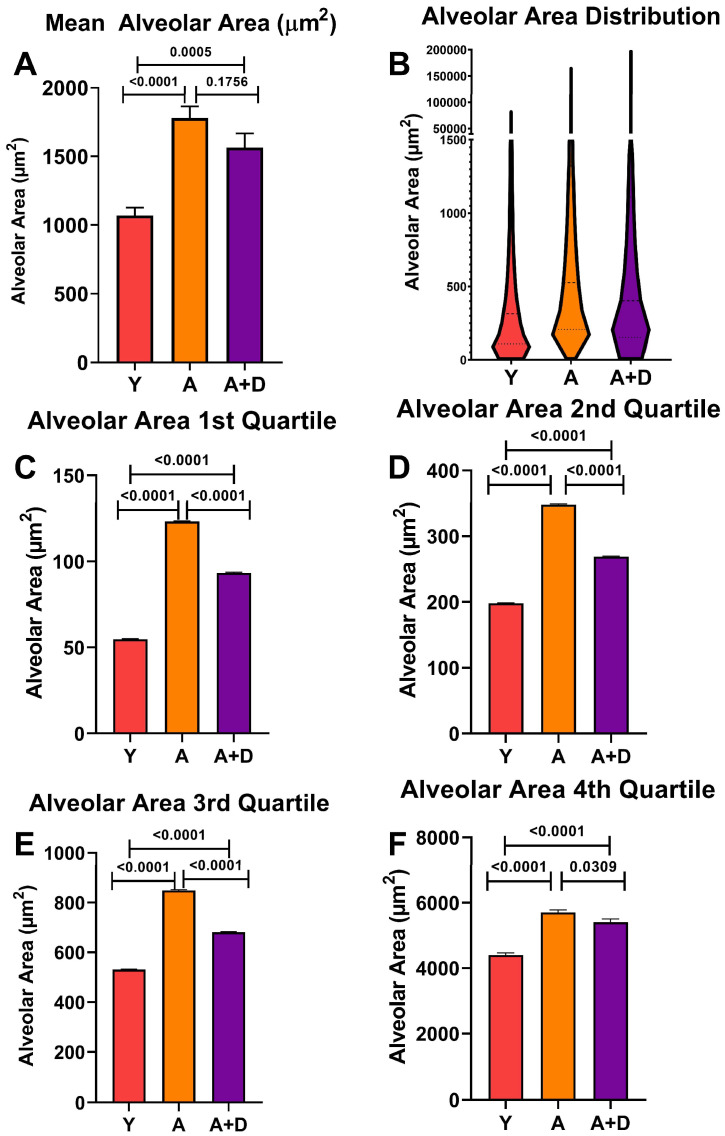
*Donepezil treatment preserves alveolar architecture*. Mean alveolar area (MAA) in young (Y), aged untreated (A), and aged donepezil-treated (A + D) mice. (**A**) Average MAA across all measured alveoli (~5000 per lung; n = 15–21 per group). (**B**) Violin plot showing distribution of individual alveolar sizes (n = 4–6 representative lungs per group). (**C**–**F**) Average MAA within each quartile of the size distribution (1st, 2nd, 3rd, and 4th quartiles, respectively; n = 4–6 per group). Bars represent mean ± SEM; *p* values shown in the figure were calculated using one-way ANOVA and Tukey’s post hoc HSD.

To assess whether donepezil affected age-related alveolar enlargement, we measured approximately 5000 alveolar spaces per lung, then averaged them per animal to find the mean alveolar area (MAA). One-way ANOVA revealed significant differences between groups (F_2,52_ = 21.0, *p* < 0.0001; Figure 4A). As expected, both aged untreated (1779 ± 390 µm^2^) and aged donepezil-treated mice (1564 ± 399 µm^2^) showed significantly increased MAA compared to young controls (1070 ± 252 µm^2^), which is consistent with age-related alveolar airspace enlargement. Notably, donepezil-treated aged mice showed a trend toward smaller MAA compared to aged untreated animals, approaching statistical significance (*p* = 0.1756).

Visual analysis of the aged lungs (Figure 3B,C) showed a wide range of alveolar sizes. The distribution of individual alveolar sizes was analyzed using a violin plot of representative lungs, shown in Figure 4B (Y: n = 4, A: n = 6, A + D: n = 6). Aged mice displayed a pronounced rightward shift in the distribution, with the increased standard deviation reflecting greater heterogeneity in alveolar sizes within Aged (SD = 4444 µm^2^) and Aged + Donepezil (SD = 5134 µm^2^) mice compared to young controls (SD = 3491 µm^2^). In contrast, observation of the mean distribution indicates that donepezil-treated aged mice maintained a substantial population of smaller alveoli alongside the age-related increase in larger alveoli, suggesting some preservation or restoration of alveolar architecture.

To quantify the possible protective effects of donepezil on alveolar size, we performed a quartile analysis of alveolar size distributions across four to six representative lungs per group (Figure 4C–F). This analysis revealed that although young mice maintained the smallest alveoli across all quartiles (*p* < 0.0001), donepezil-treated animals comparatively possessed smaller alveoli than their untreated counterparts across the entire size spectrum. In the first quartile containing the smallest alveoli (Figure 4C), donepezil-treated mice maintained significantly smaller alveoli than untreated aged animals (*p* < 0.0001), demonstrating alveolar preservation. In the middle quartiles (Figure 4D,E), donepezil-treated aged mice showed highly significant reductions in alveolar size compared to aged controls (*p* < 0.0001). Most strikingly, in the fourth quartile containing the largest and presumably most damaged alveoli, donepezil-treated mice had a significantly reduced mean alveolar size compared to aged controls (*p* < 0.05; Figure 4F), indicating that cholinergic enhancement has the potential to protect or repair even the most severely enlarged alveolar spaces. The consistent size reduction across the entire distribution demonstrates that enhanced cholinergic signaling attenuates alveolar destruction or induces pulmonary remodeling in the aged lung.

#### 3.3.2. Donepezil Treatment Preserves Elastic Fiber Content in Aged Lungs

**Figure 5:**
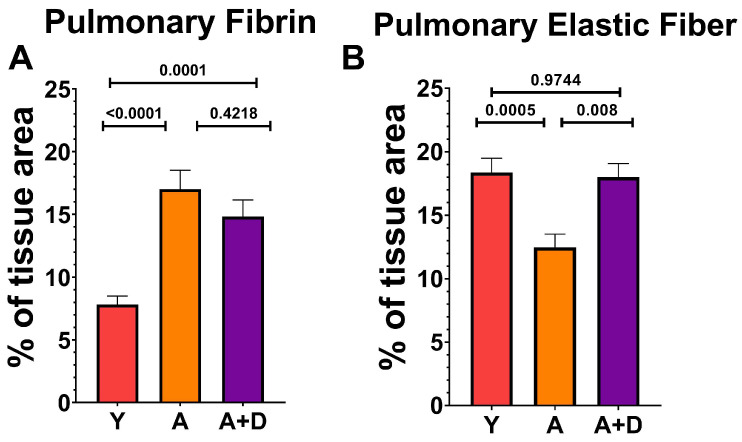
*Donepezil treatment restores pulmonary elastic fiber content*. Quantification of pulmonary extracellular matrix components in young (Y), aged (A), and aged donepezil-treated (A + D) animals (n = 15–20 mice per group). (**A**) Fibrin deposition (% tissue area). (**B**) Elastic fiber content (% tissue area). Bars represent mean ± SEM; *p* values shown in the figure were calculated using one-way ANOVA and Tukey’s post hoc HSD.

In order to examine the composition of the extracellular matrix, we quantified fibrin and elastin on Modified Russell Movat Pentachrome-stained sections (Figure 5). One-way ANOVA revealed significant differences in fibrin deposition between groups (F_2,43_ = 22.5, *p* < 0.0001; Figure 5A). As expected, both aged untreated (17.0 ± 5.4%) and aged donepezil-treated mice (14.8 ± 4.4%) showed significantly elevated fibrin compared to young controls (7.8 ± 3.1%), with no difference between the two aged cohorts. This age-related fibrin accumulation likely reflects chronic low-grade tissue injury and repair processes. In contrast, the elastic fiber content showed a remarkable pattern of distribution (F_2,54_ = 9.3, *p* < 0.001; Figure 5B). Aged untreated mice exhibited substantial loss of elastic fibers (12.5 ± 4.8%) compared to young controls (18.4 ± 5.4%), consistent with age-related elastin degradation that contributes to loss of lung compliance. Strikingly, donepezil-treated aged mice maintained elastic fiber content (18.0 ± 3.7%) that was indistinguishable from that of young animals and significantly higher than that of aged untreated controls. This complete preservation of elastic fiber architecture suggests that enhanced cholinergic signaling may protect against or reverse age-related elastin degradation, potentially maintaining the structural integrity and mechanical properties of the aging lung.

### 3.4. Donepezil Treatment Increases Total iBALT Without Inducing Pulmonary Fibrosis

**Figure 6:**
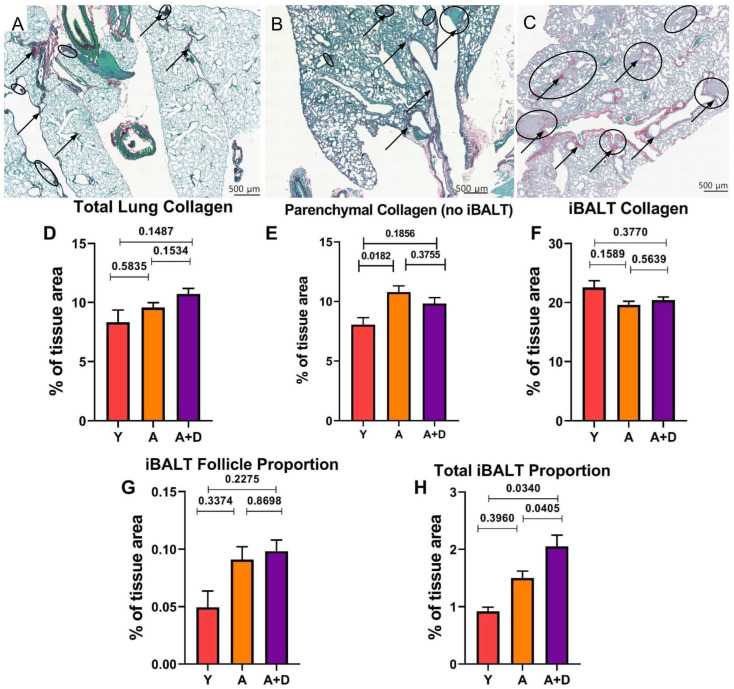
*Donepezil treatment increases iBALT*. Representative histology of Picrosirius red (PSR)-stained lung sections from (**A**) young, (**B**) aged untreated, and (**C**) aged donepezil-treated mice. Black arrows indicate collagen; black circles highlight iBALT structures. (**D**) Total collagen (% tissue area) across the entire lung. (**E**) Parenchymal collagen, excluding iBALT. (**F**) Collagen density within iBALT structures. (**G**) Average individual iBALT follicle size (% of total tissue area). (**H**) Total iBALT area per lung (% of total tissue area). Groups: young (Y, n = 6), aged (A, n = 38), and aged donepezil-treated (A + D, n = 31). Bars represent mean ± SEM; *p* values shown in the figure were calculated using one-way ANOVA and Tukey’s post hoc HSD.

Acetylcholine signaling has been implicated in TGF-b-associated tissue remodeling pathways that could promote fibrosis [62,63]. To assess whether chronic donepezil treatment adversely affects lung architecture, we quantified collagen deposition using Picrosirius red and fast green (PSR) staining, which provides superior sensitivity for collagen detection compared to pentachromic staining. Representative images, shown in Figure 6, demonstrate minimal collagen deposition (black arrows) and sparse iBALT in young lungs (Figure 6A), age-related alveolar enlargement with larger iBALT follicles (circled) in aged untreated mice (Figure 6B), and prominent collagen-rich iBALT structures in aged donepezil-treated lungs (Figure 6C).

Collagen tissue percentage was found to change with donepezil treatment, approaching statistical significance. Donepezil-treated aged mice had an increased total collagen tissue area percentage (10.72 ± 0.47%) compared to both young (8.17 ± 0.98%) and aged untreated animals (9.57 ± 0.41%; F_2,93_ = 2.78, *p* = 0.067; Figure 6D). Visually, there was an obvious increase in iBALT structures in the aged animals. This raised the question of whether donepezil was inducing pathological fibrosis or if the increased collagen reflected changes in iBALT content. To differentiate between these possibilities, we separately quantified collagen in lung parenchyma (excluding iBALT) versus within iBALT structures themselves. iBALT was assessed morphometrically, and the tissue was not immunophenotypically validated.

Analysis of parenchymal collagen (Figure 6E) indicated a significant difference was detected between the groups (F_2,64_ = 4.020, *p* = 0.0227). Aged untreated mice had elevated collagen (10.77 ± 0.53%) compared to young controls (8.05 ± 0.59%), consistent with age-related tissue remodeling. The donepezil-treated aged mice showed intermediate parenchymal collagen (9.82 ± 0.50%) that did not differ significantly from either the young or aged untreated groups. There were also differences in collagen within iBALT structures (F_2,85_ = 1.876, *p* = 0.1595). Young mice showed a higher iBALT collagen density (23.03 ± 0.71%) compared to both aged untreated (19.6 ± 0.63%) and aged donepezil-treated animals (20.4 ± 0.52%; Figure 6F).

The lack of increased collagen density within individual iBALT structures in donepezil-treated mice suggested that the elevated whole-lung collagen stemmed from an overall increase in the amount of collagen-rich iBALT tissue. To test this, we measured both the average iBALT size per mouse and total tissue area occupied by iBALT structures per mouse. The average size of individual iBALT structures increased with age but was not affected by donepezil treatment (young: 0.0494 ± 0.0142%; aged untreated: 0.098 ± 0.0112%; aged + donepezil: 0.0983 ± 0.0096%; F_2,90_ = 1.38, *p* = 0.26; Figure 6G). However, the total iBALT area as a percentage of lung tissue revealed significant differences (F_2,90_ = 5.65, *p* = 0.005; Figure 6H). Young mice had minimal iBALT (0.919 ± 0.072%), whereas aged untreated mice showed age-associated increases (1.45 ± 0.115%). The donepezil-treated aged mice exhibited a significantly greater total iBALT than either group (2.05 ± 0.196%).

Together, these findings demonstrate that enhanced cholinergic signaling via donepezil increases the total amount of iBALT in aged lungs without inducing parenchymal fibrosis, either inducing new iBALT structures or altering the iBALT collagen composition. The results also suggest that ACh plays a role in maintaining or potentially increasing the presence of existing mucosal lymphoid tissue. This represents the first evidence that acetylcholine signaling actively promotes the retention of tertiary lymphoid structures in the aging lung.

## 4. Discussion

Pulmonary aging represents a multifaceted process that is characterized by diminished oxygen absorption due to alveolar degradation following senile emphysema, loss of elastic fibrils, and pathological remodeling of respiratory tissue composition. Inflammaging is a key factor in this cumulative tissue damage [64]. ACh is the primary regulator of immune-mediated inflammation. Production of and responses to ACh decrease with aging, corresponding to the rise of inflammaging. This suggests the effects of inflammaging could be mitigated by improving cholinergic tone via increased ACh availability. We previously showed that donepezil treatment improved the lung elastic modulus of aged mice and improved lung elasticity [65]. The present study confirms and extends those findings, showing that enhancing ACh availability facilitates protective remodeling and counteracts specific pathological processes associated with age-related pulmonary decline. Aged animals receiving donepezil treatment showed significantly decreased MAA, restoration of elastic fiber content to young levels, improved SpO_2_, and increased spontaneous activity. Remarkably, donepezil treatment increased the iBALT content through the frequency of the tissue rather than the increased size. This is a completely novel finding, indicating that augmenting local ACh availability could play a role in maintaining the critical tissue-resident memory niche. Together, these functional and structural improvements underscore the cholinergic system as a clinically relevant therapeutic target for restoring pulmonary function and augmenting respiratory immune protection in the aged. These findings add to the growing body of literature calling for therapeutic intervention in the cholinergic pathway to treat disorders of aging and respiratory health [28,66,67,68,69,70,71].

The non-invasive measures of voluntary activity and blood oxygen gave the first indication that donepezil therapy improved overall health and lung function. The VWR assay has been used as a non-invasive measure of health for well over 100 years [72,73]. Voluntary activity is a similarly robust measure of overall health in humans, one that is recapitulated in the aged C57Bl/6 mice used in these studies [74,75].

Human studies have shown that AChE inhibitor therapy improves mobility and reduces inflammation-associated pain, though these studies involved Alzheimer’s patients rather than healthy aging populations [76,77,78,79,80,81,82,83,84]. The improved SpO_2_ associated with donepezil treatment has important implications for human health span, as low oxygen saturation independently predicts increased all-cause mortality and mortality from pulmonary disease in aging populations [85,86].

Donepezil-treated animals demonstrated a trend of significantly decreased MAA compared to aged controls, which suggests that treatment led to a reduction in senile emphysema, a hallmark of pulmonary aging that reflects chronic inflammatory damage to alveolar architecture [87]. Although stereological methods of airspace measurement were not employed in this study, morphological analysis portrayed a consistent trend between groups that supports our hypotheses. The results suggest that smaller alveoli are being protected or regenerated (or both) in response to donepezil treatment. The differences found between the groups’ mean SpO_2_ could, therefore, be related to this process of protection or repair, as this alveolar reinforcement would support more efficient respiration compared to aged controls. In addition, we have previously shown that altering cholinergic tone impacts pulmonary repair after influenza infection [88]. The current findings further support the idea that enhanced cholinergic signaling may be integral to interrupting inflammatory feedback loops that otherwise lead to progressive tissue damage, or in enhancing repair of the aged pulmonary environment. Additional analysis time points during and after donepezil treatment would be pivotal to identifying and assessing the mechanistic impacts of cholinergic treatment in the aged lung.

The near-complete restoration of elastic fiber content in aged donepezil-treated lungs is particularly striking, given that elastin has a half-life of approximately 70 years, and less than 1% of total body elastin turns over annually [89,90]. One likely reason for our results is reduced proteolytic degradation. Acetylcholine signaling via α7-nAChR shifts macrophage polarization from pro-inflammatory M1 toward reparative M2 phenotypes [91]. This suppresses the macrophage production of matrix metalloproteinases (MMPs), particularly MMP-9 and MMP-12, both of which mediate elastin degradation in aged lungs [92,93]. Cholinergic stimulation inhibits MMP-9 and MMP-12 production [93]. Chronic inflammation also fragments elastic fibers into elastokines, which act to perpetuate inflammatory signaling. Decreasing inflammation through increased cholinergic signaling may allow existing fragmented elastin to be reorganized or cross-linked more effectively, resulting in measurably increased elastic fiber content in the absence of de novo synthesis [94]. Finally, altering macrophage polarization from pro-inflammatory M1 toward reparative M2 phenotypes reduces TNF-α while increasing TGF-β1, potentially creating a permissive environment for elastin synthesis. TNF directly represses tropoelastin transcription and inhibits TGF-β-induced tropoelastin mRNA expression. Conversely, TGF-β1 enhances elastin expression through multiple mechanisms. These include increasing tropoelastin transcription, stabilizing tropoelastin mRNA, and promoting tropoelastin cross-linking into mature elastin fibers [95,96,97,98]. Future studies measuring MMP activity, macrophage polarization states, biomarkers of elastin degradation, and tropoelastin expression will be important to determine to what extent our findings reflect primarily reduced degradation, reorganization of fragmented elastic fibers, or enhanced synthesis.

The improved SpO_2_ in donepezil-treated mice directly addresses one of the most clinically relevant consequences of pulmonary aging and is consistent with our structural findings [99]. There is extensive literature support showing that inhibiting cholinergic signaling impacts lung remodeling, whereas increasing ACh signaling decreases acute inflammation, normalizes tissue repair, and improves lung function [44,45,46,88,92,100,101,102]. This supports our hypothesis that declining ACh availability represents a key mechanism of inflammaging and that increasing cholinergic signaling can prevent deleterious consequences of aging, including respiratory deficiency. The mechanisms underlying these improvements align with the α7 nicotinic acetylcholine receptor (α7-nAChR) pathway [42,103,104]. Enhanced ACh availability is known to promote alternative macrophage activation via α7-nAChR signaling, leading to decreased NF-κB activation and reduced pro-inflammatory cytokine production [105]. This shift from pro-inflammatory toward anti-inflammatory reparative macrophage phenotypes facilitates efferocytosis and tissue regeneration, directly addressing the dysfunctional repair mechanisms that characterize the aged lung. Engagement of this pathway was not measured directly, but the downstream effects are consistent with the trends of pulmonary improvement found between aged control and donepezil-treated lungs.

Engagement of the a7-nAChR is also associated with bleomycin-induced pulmonary fibrosis, leading us to analyze collagen deposition [63]. We did not find any evidence that donepezil treatment led to fibrotic sequela; in fact, the untreated aged animals exhibited the most lung collagen. Rather than inducing fibrosis, increasing ACh availability may actively reduce ongoing fibrotic activity or reverse fibrotic damage, potentially through tissue repair mechanisms or alternative activation of pulmonary macrophages.

### Donepezil Treatment Increases iBALT Formation: Implications for Respiratory Immunity

This study also showed that improved ACh leads to an increase in the presence of iBALT in the lung. Donepezil treatment led to a dramatically increased frequency of iBALT formation but did not necessarily expand the size of an average iBALT follicle, suggesting that ACh influences the development and maintenance of pulmonary adaptive immune capacity. This represents a previously unknown role for the non-neuronal cholinergic system in tissue-specific immune function, which could have profound therapeutic implications. iBALT serves as a site for antigen presentation, lymphocyte activation, memory cell development, and tissue-resident memory lymphocyte sequestration [106,107]. Immune responses originating from iBALT tissue-resident memory cells induce rapid local protection against infection while producing less inflammation than immune responses originating from local lymph nodes [108]. This decreased inflammation is likely due to the presence of tissue-resident memory cholinergic lymphocytes retained in the iBALT, producing local ACh as part of the secondary immune response [88,109]. The age-related loss of iBALT structures would, therefore, impair respiratory immune function through loss of local memory populations and diminished anti-inflammatory capacity. Decreased ACh availability results in extended inflammation and abnormal tissue repair after respiratory viral infection, similar to the extended morbidity exhibited by the elderly [88]. These findings support the possibility of an additional role for non-neuronal cholinergic function in respiratory immune protection. Collectively, these findings are consistent with the greatly increased susceptibility to respiratory infections associated with aging. Elevated total iBALT was shown to increase with donepezil treatment without an increase in mean iBALT size, as shown in Figure 6. As stated previously, this is an indication that iBALT frequency is increasing after treatment. This indicated that ACh availability plays a role in the initiation and/or maintenance of iBALT structures. Future studies examining the role of acetylcholine in iBALT generation and maintenance, as well as the role of cholinergic lymphocytes and the impact of cholinergic tone on respiratory immunity, will be important.

There are substantial clinical implications for our finding that donepezil treatment is associated with an increase in iBALT. Respiratory infections remain among the top causes of death in those over age 65. We acknowledge that morphological identification alone cannot fully characterize iBALT functional capacity, and that we have yet to confirm if there is a therapeutic benefit to increased ACh-induced iBALT. iBALT can be either protective or pathogenic depending on context, cellular composition, and the balance of regulatory versus effector populations [39,107]. However, our finding that cholinergic enhancement strengthens the lung’s capacity for local adaptive immunity is consistent with clinical studies showing that donepezil treatment reduces the risk of death from respiratory infection and supports the concept that increasing iBALT capacity is associated with beneficial outcomes for a respiratory infection [45,46]. Our use of donepezil, an FDA-approved medication with an established safety profile in elderly patient populations, significantly enhances the translational relevance of our study. Retrospective analyses have demonstrated that donepezil treatment is associated with a reduced overall mortality and pneumonia-related mortality [45,71,110]. Furthermore, enhancing the respiratory immune response through iBALT formation suggests potential applications beyond simple anti-aging interventions, including the enhancement of vaccine efficacy and respiratory infection resistance in elderly populations.

This current study suggests that ACh acts as an autocrine/paracrine signal in iBALT retention. While this study did not identify the specific source(s) of pulmonary ACh production, our previous study indicated that cholinergic lymphocytes were retained in iBALT after respiratory infection, making them a likely source of immunomodulatory ACh [88]. However, the lung has multiple other ACh sources, including cholinergic neurons and bronchial epithelium. Critical next steps include the development of specific biomarkers for cholinergic lymphocytes to enable their isolation and characterization in human studies, as well as a direct measurement of tissue ACh levels and AChE inhibition, and an evaluation of receptor dependence using receptor-deficient models. Additionally, future assessment of inflammatory mediators, macrophage polarization states, and the iBALT cellular composition and role in protective immune function will provide deeper mechanistic insights into donepezil’s role in the cholinergic system, both in the pulmonary system and systemically.

A key limitation of this study is that enhanced pulmonary acetylcholine signaling is inferred from the known mechanism of donepezil and the observed phenotypic changes. ACh bioavailability and cholinergic receptor expression and function were not directly measured. However, our findings that donepezil treatment improved aged lung structure and function are consistent with studies showing the negative impact of decreased ACh availability on lung tissue as well as the survival benefits associated with donepezil treatment during respiratory infection [45,71,88,92]. Future studies will include confirmation of altered ACh bioavailability as well as the role of specific cholinergic populations and characterization of AChR-mediated pathways (particularly α7-nAChR signaling) underlying the observed improvements. Upcoming experiments could utilize genetic knockout mice or direct measures of ACh to directly measure increases in cholinergic tone and ensure treatment is directly affecting the pulmonary system. Moreover, the lack of mid-treatment timepoints obfuscate potential differences between treatment arresting ongoing damage versus the possible induction of tissue repair. Finally, our investigations of MAA and iBALT were based on morphometric observations of pulmonary histology rather than the more powerful stereometric or immunophenotypic analyses, respectively. Future studies will address these mechanistic questions and include detailed characterizations of cholinergic lymphocyte function and local inflammatory parameters.

One key strength of this study is the aged murine model system. Employing an aged mouse model is a significant advantage, as murine pulmonary, cholinergic, and immunological aging closely parallels human systems. The use of naturally aged mice allowed us to evaluate the therapeutic impact of increased ACh bioavailability in a physiologically relevant model of human pulmonary aging. Furthermore, although acetylcholine concentrations also vary across species, fundamental cholinergic mechanisms regulating immune function appear evolutionarily conserved [111]. Although we did not detect any sex-specific differences associated with donepezil treatment, sex hormones are known to modulate both immune function and cholinergic signaling [112,113,114]. The immune system also ages differently between sexes, with males showing increased pro-inflammatory background and greater age-related changes in immune function [112,115]. Species differences in cholinergic system anatomy and function also merit consideration. However, murine cholinergic aging closely parallels human systems; absolute acetylcholine levels, receptor expression patterns, and acetylcholinesterase isoform distribution differ between species [116,117]. However, donepezil’s established safety profile and existing clinical use in elderly humans provide a direct translational bridge. Retrospective clinical studies showing a reduced mortality from respiratory infections in donepezil-treated patients support the clinical relevance of our mechanistic findings despite potential species differences in the absolute magnitude of effect [45,46]. These considerations underscore both the need for additional preclinical validation across diverse models and the importance of prospective clinical trials examining respiratory changes in elderly populations receiving acetylcholinesterase inhibitor therapy.

## 5. Conclusions

Our results demonstrate that the non-neuronal cholinergic system represents a critical therapeutic target to reverse age-related pulmonary damage and enhance tissue-specific adaptive immune function. The comprehensive improvements observed with donepezil treatment included a partial reversal of alveolar enlargement, restoration of elastic fiber content, enhanced blood oxygenation, and augmented adaptive immunity through iBALT retention. This provides compelling evidence that age-related pulmonary decline results from cholinergic dysfunction. In addition, our discovery that cholinergic enhancement dramatically increases iBALT formation reveals a previously unrecognized mechanism by which ACh availability influences adaptive immune capacity in the aged lung. These findings support a therapeutic paradigm whereby improving cholinergic tone could reverse multiple aspects of age-related respiratory decline while augmenting respiratory immune function.

The clinical translatability of these findings is straightforward, given the known safety profile and existing clinical use of acetylcholinesterase antagonists in elderly populations. Retrospective clinical analysis confirms that donepezil reduces mortality from respiratory infection, further supporting the therapeutic potential of our study. Future clinical studies examining respiratory function and infection outcomes in the context of cholinergic enhancement will determine whether targeting the non-neuronal cholinergic system can translate from a promising experimental intervention to a clinically meaningful approach for extending one’s respiratory health span and reducing age-related infection susceptibility.

## Data Availability

The raw data supporting the conclusions of this article will be made available by the authors on request.

## Abbreviations

The following abbreviations are used in this manuscript:

ECM	Extracellular matrix
ACh	Acetylcholine
CAP	Cholinergic Anti-Inflammatory Pathway
AChE	Acetylcholinesterase
iBALT	Induced Bronchus-associated lymphoid tissue
IACUC	Institutional Animal Care and Use Committee
VWR	Voluntary wheel running
LHS	Lung and Heart sound (monitor)
SpO_2_	Peripheral oxygen saturation
MAA	Mean alveolar area
ANOVA	Analysis of variance
BSAF	Biebrich scarlet-acid fuchsin
PSR	Picrosirius red
α7-nAChR	α7 nicotinic acetylcholine receptor

## Appendix A

### Expanded Methods

Image Analysis: The stained slides were imaged utilizing the SDSU EM Facility’s BZ-X810 Fluorescence Microscope (Keyence Corp., Osaka, Japan). The images were captured with the 10× lens (100× total power), with the exposure set to 1/800. Boundaries of the tissue samples were identified, and then the microscope performed a tile scan. The image tiles were arranged according to the program’s logic, then combined into a large image file of the total tissue sample for analysis.

After capture, the images were edited in the BZ-X800E analyzer software (version BZ-H4C) (Keyence Corporation, Osaka, Japan) to add a 500-micron scale bar for ease of comparison. To find a sample’s mean alveolar area, the alveolar wall tissue was identified using the Keyence Hybrid Cell Count software. Then, the inversion function was used, falsely coloring all open spaces within the lung (Figure A1). The inverted images had vasculature, bronchi, and bronchioles manually excluded, ensuring only the alveolar space was measured. Finally, the area from each alveolar space was tabulated in square microns, averaged, and then saved as a “.csv.”

To investigate the proportion of lung tissue stained by each histological treatment, the images generated above were processed through a separate feature of the Keyence Hybrid Cell counter, termed a “Single Extraction.” Alveolar septal tissue was identified and selected, and the tissue underwent a second round of selection: the “Single Extraction.” From the previously selected tissue, the hybrid cell counter enters another round of analysis, bringing up the hue-selection toolbar. The hue toolbar was used to identify pentachromic-stained components of the ECM via color identification. Pixels of the color of interest were selected manually from the image. The selection was guided by the appearance of the positive control. The program highlighted pixels that matched the specified color and added them to the end analysis. Multiple pixel selections were required per image. Finally, the program compared the proportion of selected or “extracted” colors to the initial total tissue area. This is defined as the “Mean Extraction Percentage.” These calculations were exported in a “.csv” file. Each total-lung image was analyzed for the mean extraction percentage of the stain, which represents the percentage of the tissue stained. Thresholds for segmentation and color selection were applied uniformly across cohorts; the extracted pixel area is reported as the percentage of tissue area (%). Section-level sampling was balanced across cohorts, and section analysis was averaged per individual animal. Identification of iBALT was based on morphology and location; immunophenotypic confirmation was not performed.
